# A systematic review and meta-analysis of the hip capsule innervation and its clinical implications

**DOI:** 10.1038/s41598-021-84345-z

**Published:** 2021-03-05

**Authors:** Joanna Tomlinson, Benjamin Ondruschka, Torsten Prietzel, Johann Zwirner, Niels Hammer

**Affiliations:** 1 Department of Anatomy, School of Biomedical Sciences, University of Otago, Dunedin, Otago New Zealand; 2 Institute of Legal Medicine, University Medical Centre Hamburg-Eppendorf, Hamburg, Germany; 3 Department of Orthopaedics, Trauma and Reconstructive Surgery, Zeisigwaldkliniken Bethanien, Chemnitz, Saxony Germany; 4 Institute of Macroscopic and Clinical Anatomy, University of Graz, Graz, Styria Austria; 5 Department of Orthopaedic, Trauma and Plastic Surgery, University of Leipzig, Leipzig, Saxony Germany; 6 Division of Medical Technology, Fraunhofer Institute for Machine Tools and Forming Technology (Fraunhofer IWU), Dresden, Saxony Germany

**Keywords:** Anatomy, Musculoskeletal system, Nervous system

## Abstract

Detailed understanding of the innervation of the hip capsule (HC) helps inform surgeons’ and anaesthetists’ clinical practice. Post-interventional pain following radiofrequency nerve ablation (RFA) and dislocation following total hip arthroplasty (THA) remain poorly understood, highlighting the need for more knowledge on the topic. This systematic review and meta-analysis focuses on gross anatomical studies investigating HC innervation. The main outcomes were defined as the prevalence, course, density and distribution of the nerves innervating the HC and changes according to demographic variables. HC innervation is highly variable; its primary nerve supply seems to be from the nerve to quadratus femoris and obturator nerve. Many articular branches originated from muscular branches of the lumbosacral plexus. It remains unclear whether demographic or anthropometric variables may help predict potential differences in HC innervation. Consequently, primary targets for RFA should be the anterior inferomedial aspect of the HC. For THA performed on non-risk patients, the posterior approach with capsular repair appears to be most appropriate with the lowest risk of articular nerve damage. Care should also be taken to avoid damaging vessels and muscles of the hip joint. Further investigation is required to form a coherent map of HC innervation, utilizing combined gross and histological investigation.

## Introduction

When performing total hip arthroplasty (THA), one philosophy of orthopedic surgeons aiming to preserve and repair the hip joint capsule (HC) is to spare the surrounding muscles and surrounding tissue, in turn this may also spare the articular branches of nerves supplying the HC^1,2^. Conversely, anesthetists and pain physicians aim to target these structures for radiofrequency ablation (RFA) procedures when THA is contraindicated, such as in patients with severe comorbidity, THA is deemed inappropriate, or for patients who require RFA as a temporary measure^3^. These clinicians rely on the spatial anatomy of the hip joint and its innervation patterns to inform their practice. However, in spite of high success rates for both procedures, postprocedural persistent pain is prevalent^4,5^. Specifically, pain following RFA^3,4^ and dislocation rates following THA^6,7^ could be reduced. It is unclear if persistent pain following RFA is due to inaccuracy when approaching the nerve’s articular branches due to lacking detailed anatomical knowledge, insufficient RFA resulting from the size or number of radiofrequency lesions, destruction of the nerve architecture or other factors^3^. A reduction in dislocation rate following THA appears achievable by repairing the capsule^8–23^, it has been theorized that this may partly be a result of preservation proprioceptive functioning^8–10,24^, in addition to restoration of the biomechanical properties of the HC. Proprioception of the hip joint; which is the conscious and unconscious sensation of the body in space^25^; is thought to be controlled by the end-terminals of the nerves to the HC^1^, as well as receptors present in the skin and musculature acting on the joint^26,27^. To date, it is unclear to what extent the neural feedback of the HC contributes to the success of THA with capsular repair.

The posterior [Kocher–Langenbeck] and lateral [Bauer or Hardinge] approaches appear to be the most commonly used methods during THA^28^. The choice of the surgical approach is often based on the surgeons’ educational environment, consequential experience and preference following good outcomes^29^. Surgeons focus on preserving muscular integrity^30–34^ and ensuring correct alignment of the implant^9,35^ in order to reduce risk of dislocation, rather than placing emphasis on assessing the patient’s peripheral neuroanatomy, which remains obscure when planning the surgery in the first instance. There is mixed evidence on the contribution of surgical approach to dislocation and instability^36–41^. Historically most studies report increased dislocation rate via the posterior approach^39–43^, however, when pooling data the risk appears to be similar^38^ and is further reduced when repair of the capsule and surrounding musculature is performed^20,21^. This indicates that other factors are likely to play a role in stability and an individualized approach which accounts for the patient’s anatomy may be required. A recent review of microscopic studies has shown that the highest number of nerve endings (type I-III mechanoreceptors and free nerve endings (FNE) are generally present in the proximal and superior lateral aspect of the HC, suggesting this region should be avoided during THA^44^ in order to maintain proprioceptive functioning. This region may be a suitable target for RFA to reduce pain, however, the anteromedial aspect of the HC is commonly used^4^. It is unclear if these microscopic findings corroborate with macroscopic studies and the extent of differences in innervation between individuals. The origin of these nerves also remains unclear, Hilton’s law suggests that any nerve supplying a muscle crossing the joint also innervates the inner aspects of the joint^45^; it is however uncertain if this also applies to the HC. It is also unclear if avoiding damage to these nerves is sufficient to prevent dislocation of the joint.

### Aims and objectives

This systematic review and meta-analysis aims to provide an encompassing synthesis of contemporary literature on the HC’s innervation from gross anatomical studies. This includes additional analyses that have not been performed to date^4,46^: a meta-analysis of the prevalence of nerves innervating the HC, discussion of the course, density, and distribution of articular nerves, and changes according to demographic, arthropometric variables and underlying pathology.

## Methods

### Selection of studies

A systematic review of the literature on the HC’s innervation was performed to identify peer-reviewed articles published until October 2020 according to the Preferred Reporting Items for Systematic Reviews and Meta-analyses (PRISMA) guidelines^47^. Keywords relating to the HC’s innervation in gross anatomical studies (macroscopic studies) were searched using the following online databases: Amed (1985 <), Embase (1947 <), and Medline (1949 <) via Ovid, also PubMed, ScienceDirect, Scopus and Web of Science (all approximately 1900 <). Search terms are outlined in Table 1. Wiley and SpringerLink electronic databases were also screened ensure articles published in relevant anatomical journals from a wider date range were searched. The reference lists of previous literature reviews^4,46^ and the articles identified in this search were screened. To locate relevant articles that cite the included articles Web of Science Core Collection and ResearchGate.net were screened.

**Table 1:** Search terms used in search strategy for this systematic review. Using the Boolean search method, the search lines were combined with the word ‘AND’. *Truncation of a word, this searches for words that begin with the word preceding the asterisk.

Search combinations
**(A) AND (B) AND (C)**
**Search term (lines)**
(1) acetabulofemoral (2) coxa (3) hip
**(A) (1) OR (2) OR (3)**
(4) capsul* (5) iliofemoral (6) ischiofemoral (7) ligament* (8) pubofemoral (9) pseudocapsul*
**(B) (4) OR (5) OR (6) OR (7) OR (8) OR (9)**
(10) anatomy (11) cadaver* (12) dissect* (13) donor* (14) innervat* (15) morphology (16) nerve
**(C) (10) OR (11) OR (12) OR (13) (14) OR (15) OR (16)**

When the databases included search options, these were configured to remove non-primary research articles and duplicates. Articles were inputted into an Excel spreadsheet to remove duplicates, then were screened by one author (J.T.) by their title and abstract, then by their full text. All screened articles that included macroscopic study of the HC or surrounding structures (i.e., musculature, bones and vessels) from either surgical or cadaveric studies were reviewed in their full text, to ensure all incidental findings on the HC’s innervation were included. The following exclusion criteria were applied during the literature search:

- Animal studies
- Non-peer reviewed articles
- Non-primary research articles
- Non-macroscopic studies
- Studies that did not investigate the HC or associated structures

When an abstract was absent the full text was evaluated. Papers were included in the review if they contained specific information on the HC’s macroscopic innervation. Selected studies were assessed to ensure duplicated information was not included in this review.

### Data extraction

Relevant data was extracted from studies by one author (J.T.), this included information on the sample size investigated, demographic information of samples studied (age, sex, ethnicity, side and weight), as well as details on the prevalence, origin, course, and distribution of innervation.

### Critique of the literature

Papers were critiqued by one researcher (J.T.) according to the several criteria adapted from the AQUA^48^ and QUACS^49^ scales to assess the limitations to the conclusions made in cadaveric studies. This included assessing aspects of the methodology and potential variables affecting the reliability of the results presented, including determining if data was prospectively or retrospectively collected and if magnification was used. Additionally, the replicability of the study, data presentation and consideration of limitations were assessed.

### Analyses

The HC’s innervation was evaluated by collating information on the course and density of innervation and information on prevalence and distribution underwent analytical processing.

### Prevalence

Meta-analysis was performed to determine the pooled prevalence estimate (PPE) of each nerve innervating the HC. Analysis of the effect size was determined using a random effects model computed in Microsoft Excel 2016 with the MetaXL add-on version 5.3 (EpiGear International Pty Ltd, Queensland, Australia). The random effect model was selected as information from cadaveric samples generally have high heterogeneity^50^. By applying a random effects model this assumes a normal distribution within the sample, that the effects are estimated and different studies are not identical^51^. The PPE analysis included a double arcsine prevalence transformation, with a continuity correction of 0.5 and 95% confidence intervals (CI), in order to address the issues of variance instability^52^. The prevalence was obtained by multiplying the computed PPE by 100 to form a percentage. Several inclusion criteria were required for the PPE to be assessed:

- Studies with over 10 hip joints were investigated.
- The prevalence of a certain nerve innervating the HC was calculated when there were more than two studies investigating that nerve. More than 10 studies on the topic were regarded as a substantial number to form valid conclusions from the literature.
- Only prospective studies were included, retrospective studies were excluded as there is a risk of selection bias.

The variance between studies included in the meta-analysis was studied using the I^2^ statistic (which assesses the amount of heterogeneity between studies). The following standard percentages were used to assess variance: < 40% indicates low heterogeneity, 30–60% suggests moderate, 50–90% implies substantial and 75–100% may be considerable heterogeneity^51^. Publication bias was analysed by visually assessing the funnel plots, doi plots and Luis–Furuva–Kanamori (LFK) index^53^. No publication bias was assumed from symmetry in the LFK index and in the absence of symmetry publication bias was assumed. An LFK index within ± 1 from 0 indicates no asymmetry, out of ± 1 but within ± 2 as minor asymmetry, and >  ± 2 is to mean major asymmetry^53^.

### Distribution

The nerve distribution was assessed by collating information from the literature regarding which region of the HC was supplied by each nerve. A heat map was created for each nerve, to represent the areas of agreement of the distribution of innervation between studies. The opacity from black to white displayed in the heat map directly related to the percentage of agreement between studies, where black represents 100% agreement and white represents no agreement.

## Results

### Few papers on the hip capsule innervation were identified from the extensive search

**Figure 1:**
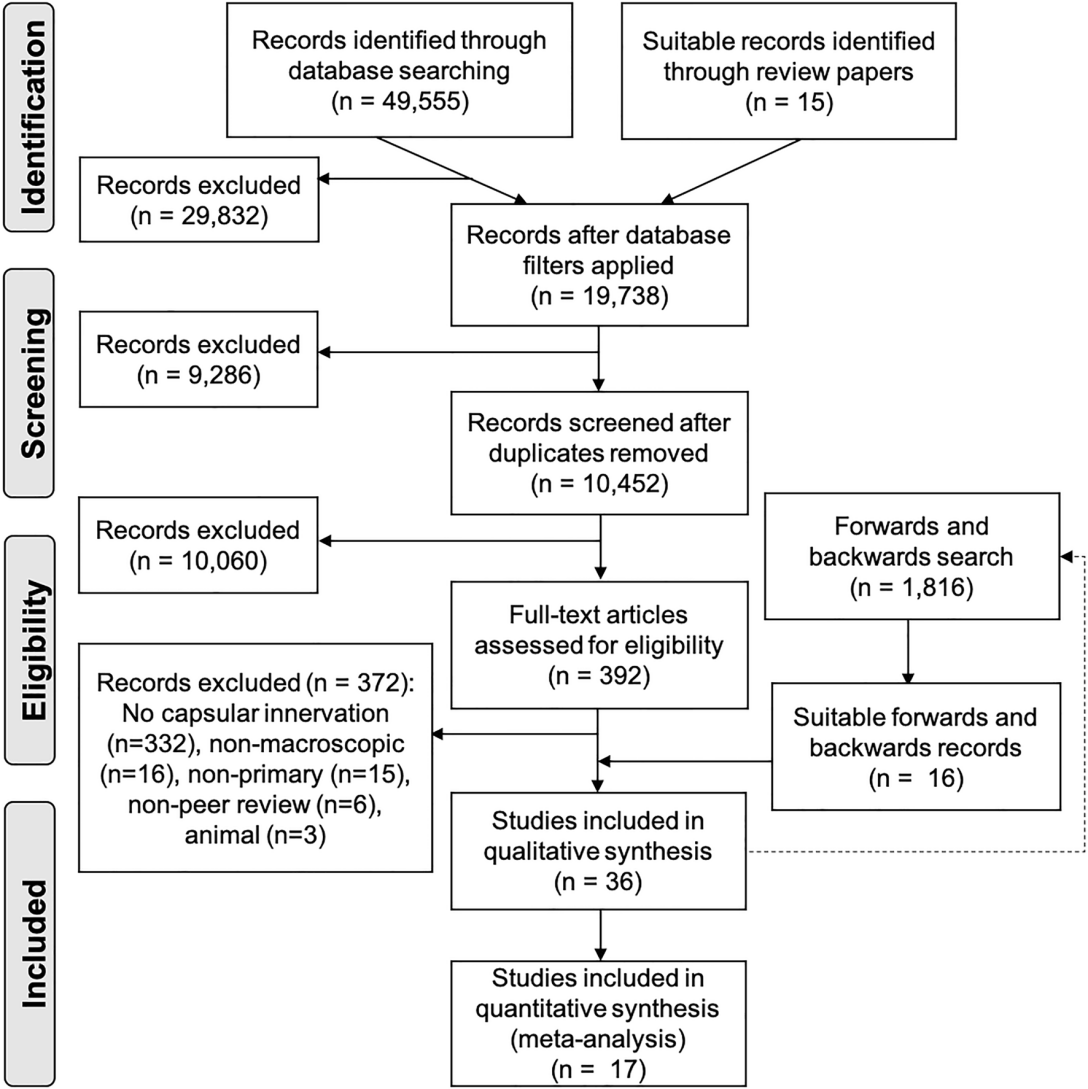
PRISMA flow chart displaying the search results based on Moher et al.(2010)^22^. The dotted line represents the articles located from the search of the databases undergoing forwards and backwards chain sampling to located further articles.

In total, 36 papers were identified as eligible for inclusion from 49,555 records overall (see Fig. 1). These were published between 1857 and 2020. Twenty-six were available in English, five were published in French^54–58^, three in German^59–61^, one in Russian^62^ and one in the Czech language^63^. Translations were performed by native speakers. Four studies potentially include duplicated results^60,63–65^. However, as they discuss different aspects of the HC innervation, they were included in the descriptive analysis but duplicates were excluded from the meta-analysis.

### Varying methodologies were employed to study hip capsule innervation

Despite all the studies including dissection of cadaveric material, the methodologies employed differed substantially and several papers provided limited information making it difficult to assess the quality of the studies included (see Table 2). Numerous authors acknowledge that factors may have affected the results presented: post-mortem changes^66,67^, their dissection^66–69^, embalming^70^, the limited sample size^65,67,71^ and the morphology of the articular nerves, which are noted to be thin and may have been damaged^56,57,62,72^. It is unclear if spatial distortion or excision of nerves during dissection affected the results^66–69^. Several authors partially accounted for potential limitations by excluding cadavers from their study based on their observations or records of the following: pathology^67,73,74^, previous spinal^67,70^, abdominal^70^ or hip surgery^67,70,73^. Others used magnification to aid their dissection^58,60,69,72,75–78^, which some authors thought this was essential when investigating this region^77^. Some studies used histological stains of capsular tissue alongside their dissection^63,79^. However, they did not clearly present their findings^63,79^.

**Table 2:** Table of the critique of the literature.

Paper	Prospective or retrospective study	Clearly defined sample (e.g., age, sex, side)	Cadavers excluded with reasons	Study type (e.g., formalin, fresh-frozen)	Use of magnification during dissection	Replicable and clearly defined methodology	Clearly defined outcomes and results	Clear diagrams and images to display the results	Justified interpretation of the results and concise conclusion	Clearly state limitations or bias of research
Berhanu et al. (2020)^71^	P	Y	N	Embalmed	N	Y	Y	Y	Y	N
Nielsen et al. 2018^66^	P	Y	N	Ethanol- glycerol and phenolic acid	NS	Y	Y	N	U	Y
Sakamoto et al. 2018^64^	P	Y	N	Embalmed	NS	Y	Y	N	Y	Y
Short et al. 2018^67^	P	Y	Y	12 formalin and 1 light embalm	NS	Y	Y	Y	Y	Y
Nielsen et al. 2017^68^	P	Y	N	Ethanol- glycerol and phenolic acid	NS	Y	Y	N	U	Y
Turgut et al. 2017^80^	P	Y	N	Fresh frozen	NS	Y	Y	Y	U	N
Sakamoto et al. 2014^65^	P	N	N	Formalin	NS	N	N	N	U	Y
Rohini et al. 2012^81^	R	N	N	NS	NS	Y	Y	N	U	N
Anagnostopoulou et al. 2009^82^	P	Y	N	NS	NS	Y	Y	N	Y	N
Anloague and Hijbregts 2009^70^	P	Y	Y	Embalmed	NS	Y	U	N	Y	Y
Apaydin et al. 2009^73^	P	Y	Y	NS	NS	Y	N	N	U	N
Locher 2008^69^	P	Y	N	Embalmed	Y	Y	Y	U	Y	N
Kampa et al. 2007^83^	P	Y	N	Formalin	NS	Y	Y	Y	Y	Y
Birnbaum et al. 2000^74^	R	Y	N	NS	NS	N	Y	N	U	N
Tubbs et al. 2003^84^	P	Y	Y	Formalin	NS	Y	Y	N	U	N
Birnbaum et al. 1997^76^	P	N	N	Formalin	Y	N	Y	Y	Y	N
Aizawa 1992^75^	P	N	N	NS	Y	N	N	N	U	N
Katrisis 1980^85^	P	N	N	Embalmed	NS	N	N	N	U	N
Dee 1969^78^	P	N	N	Fresh	Y	N	N	N	U	N
Moghaddam 1962^59^	P	N	N	NS	NS	N	N	N	U	N
Poláček 1963^60^	P	Y	N	NS	Y	N	N	Y	U	N
Woodburne 1960^86^	R	N	N	NS	NS	N	N	N	U	N
Poláček 1958^63^	P	N	N	NS	NS	N	Y	N	U	N
Wertheimer 1952^77^	P	N	N	Formalin	Y	N	Y	Y	Y	N
Gardner et al. 1948^87^	P	N	N	NS	NS	N	N	Y	U	N
Kaiser 1949^79^	P	N	N	NS	NS	N	Y	Y	U	N
Poulhés 1949^58^	R	N	N	NS	Y	N	N	Y	U	N
Tavernier and Pellanda 1949^56^	R	N	N	NS	NS	N	N	N	U	N
Kaplan 1948^72^	P	N	N	NS	Y	Y	Y	Y	Y	N
Billet et al. 1947^57^	R	N	N	NS	NS	N	N	N	N	N
Sadovsky 1933^62^	P	Y	N	NS	NS	Y	Y	N	U	N
Jamieson 1903^88^	R	N	N	NS	NS	N	N	N	U	N
Paterson 1891^89^	R	N	N	NS	NS	N	N	N	U	N
Chandelux 1886^55^	R	N	N	NS	NS	N	N	N	U	N
Duzéa 1886^54^	R	N	N	NS	NS	N	N	N	U	N
Meyer 1857^61^	R	N	N	NS	NS	N	N	N	U	N

A quality analysis of papers included in this review was performed using a modified quality assessment tool for macroscopic anatomical studies, adapted from Henry et al. (2017) and Wilke et al. (2015)^48,49^.

*N* no, *NS* not stated, *P*  prospective, *R*  retrospective, *U*  unclear, *Y*  yes.

Several factors prevent a full assessment of the quality of studies. Many studies omitted to explain their methodology^54–59,61,63,84–86,88,89^ or failed to provide sufficient information that would enable it to be replicated^60,65,75,77,78,87^. While other studies were retrospective observational analyses^54–58,61,74,81,86,88^. Furthermore, some papers did not include images of their dissection^54–62,69,72,73,75,77–79,86–89^. No paper discussed the degree of degradation of the tissue or time from death to embalming as a measure of quality control. The studies included in this review have limitations as expected from this study type, but many authors are aware of these when drawing their conclusions.

### Summary of descriptive data

### Limited information on the sample characteristics were presented in the literature

The study characteristics are presented in Table 3. All studies included in this review investigated cadaveric material. Studies included samples sizes ranging between 1–1000 (mean 92) hip joints, though seven studies did not specify the number of hip joints studied^54,55,58,59,61,62,88^. Available data in the literature was limited, as only eight studies indicated the age of the cadavers in their experiment^64,66,67,70,74,82–84^, five studies detailed the ethnicity^64,65,71,75,79^ and one study noted the average weight^82^. No other demographic or anthropometric variables were described in the studies included.

**Table 3:** Table of study characteristics including sample size and demographic information of cadaveric material studied (age, sex, side, ethnicity), in descending chronological order list. NS = not stated.

Study	Number of cadavers included	Sample size (hip joints)	Range or average age at time of death	Sex ratio (female : male)	Side ratio (right:left)	Ethnicity
Berhanu et al. (2020)^71^	34	67	NS	NS	34:33	Ethiopian
Nielsen et al. 2018^66^	8	15	81–98 years	7:1	NS	NS
Sakamoto et al. 2018^64^	14	14	73.8 years	5:9	0:14	Japanese
Short et al. 2018^67^	13	13	79.3 years	9:4	NS	NS
Nielsen et al. 2017^68^	7	14	NS	4:3	7:7	NS
Turgut et al. 2017^80^	20	40	NS	12:8	20:20	NS
Sakamoto et al. 2014^65^	2	NS	Adult	NS	NS	Japanese
Rohini et al. 2012^81^	1	1	Adult	0:1	0:1	NS
Anagnostopoulou et al. 2009^82^	84	168	72 years	30:54	84:84	NS
Anloague and Hijbregts. 2009^70^	19	38	75.1 years	9:10	19:19	NS
Apaydin et al. 2009^73^	18	36	NS	8:10	18:18	NS
Locher 2008^69^	10	14	NS	7:3	NS	NS
Kampa et al. 2007^83^	20	20	81 years	10:10	8:12	NS
Birnbaum et al. 2000^74^	NS	14	78 years	6:8	NS	NS
Tubbs et al. 2003^84^	1	2	71 years	1:0	1:1	Caucasian
Birnbaum et al. 1997^76^	NS	11	NS	NS	NS	NS
Aizawa 1992^75^	137	265	NS	NS	NS	Japanese
Katrisis 1980^85^	500	1000	Adult	158: 342	500:500	NS
Dee 1969^78^	NS	41	NS	NS	NS	NS
Moghaddam 1962^59^	3	NS	NS	NS	NS	NS
Poláček 1963^60^	NS	30	Adult and foetus	NS	NS	NS
Woodburne 1960^86^	NS	550	NS	NS	NS	NS
Poláček 1958^63^	25	30	Adult and foetus	NS	NS	NS
Wertheimer 1952^77^	NS	124	Adult	NS	NS	NS
Gardner et al. 1948^87^	NS	11	NS	NS	NS	NS
Kaiser 1949^79^	NS	24	NS	NS	NS	NS
Poulhés 1949^58^	NS	NS	Adult and new-born	NS	NS	NS
Tavernier and Pellanda 1949^56^	NS	100	NS	NS	NS	NS
Kaplan 1948^72^	NS	28	NS	NS	NS	NS
Billet et al. 1947^57^	NS	12	NS	NS	NS	NS
Sadovsky 1933^62^	30	NS	Adult	NS	NS	NS
Jamieson 1903^88^	NS	NS	NS	NS	NS	NS
Paterson 1891^89^	20	40	NS	NS	20:20	NS
Chandelux 1886^55^	NS	NS	NS	NS	NS	NS
Duzéa 1886^54^	NS	NS	NS	NS	NS	NS
Meyer 1857^61^	NS	NS	NS	NS	NS	NS

### The hip capsule receives innervation from a variety of nerves of the lumbosacral plexus and their muscular branches

The HC appears to receive innervation from articular branches of nerves of the lumbosacral plexus. Specifically, studies have reported articular branches from the femoral^54,56,57,60,62,64–67,75–78,83,87^, obturator^54,56–62,64,65,67,69,71,75–79,82,83,87^, accessory obturator (AON)^56,57,60,63,67,68,70,77,79–81,83–89^, accessory femoral (AFN)^56,60,76^, superior gluteal^60,62,74,76,83,87^, accessory superior gluteal^78^, inferior gluteal^83^, sciatic^55–57,60,62,76,77,83^, posterior femoral cutaneous nerves^72^, nerve to quadratus femoris (NQF)^57,61,76–78,87^ and a direct supply from the sacral plexus^72^. One study explicitly noted an absence of articular branches originating from the pudendal nerve^60^, however, it is important to note that their study does not confirm its absence. Other studies suggested that sympathetic fibres supply the HC, but did not prove this histologically^58,87^.

The roots of the nerves providing articular branches to the HC have only been reported for the obturator nerve, AON and AFN and are highly variable. In one study of Ethiopian cadavers, the obturator nerve originated primarily from L2,3,4 and in some cases from L3,4^71^. When the AON was present, it originated from either L2-3^80,85,88^, L2-4^85^, L2-L5^67^, L3^80,85,88,89^, L3-4^63,80,81,84–86^, from the obturator nerve^80,85^ or from the femoral nerve^80^. It is unclear which nerve root the AON most commonly originates from^80,85^. When the AFN was present its nerve roots were from L2,3^63^ or L2,3,4^75^ and therefore was more predictable.

Articular branches appear to be derived from branches to some of the muscles that act on the hip joint^56,60,62–66,75–79,85–89^ or directly from the nerve before it divides to supply the muscles^56–58,62,64,65,67,72,76–79,82,87^. However, this has not been systematically studied to date. The location of the origin from the main trunk appears to be variable and has only been recorded for the obturator and femoral nerves. Articular branches from the obturator were from the posterior branch^56,62,64,65,71,72,76,77,79,82^, the anterior branch^56,62,64,71,76,77,82,87^ and most commonly from the common obturator trunk^56,71,76–78,82,87^. These articular branches may divide from the obturator nerve at several levels: proximal to the canal within the pelvis^77,79^, at the internal orifice^77,79^, within the canal^56–58,77,79^ or at the external orifice^57,77,79^. It is unclear if articular branches of the obturator nerve are most commonly derived from a high, low origin or both^67^. Whereas, articular branches of the femoral nerve more commonly originated from a high branch rather than from a low branch^67,81^.

### Information on the course of hip capsule’s articular branches is sparse

The course of the HC’s articular branches was described by few authors and lack a uniform description of their course in relation to the muscles, osseous landmarks, or vessels. Articular branches from the femoral^58,76,77^, obturator^58^ and superior gluteal^83^ nerves were described as coursing with vessels. Specifically, these articular branches coursed deep to the femoral artery^58^, also along the obturator^58^, the lateral circumflex^58^ and posterior circumflex arteries^58,63^. However, this has not been assessed systematically. Based on the literature, articular branches appear to cross all regions of the HC^57,58,64,78,83,87^. Some branches have a long course and do not terminate proximal to where the main trunk overlies the capsule, for example some articular branches from the obturator and femoral nerve coursed posteriorly^55,58,63,76^. To date, no study has described the course of the AON, AFN, sciatic or inferior gluteal nerves in detail. Articular branches appear to course indirectly across the anterior and posterior HC to their point of termination, but the current literature remains unclear with regards to the specific location of these nerves.

### Hip capsule innervation from the obturator nerve and nerve to quadratus femoris is most prevalent

**Figure 2:**
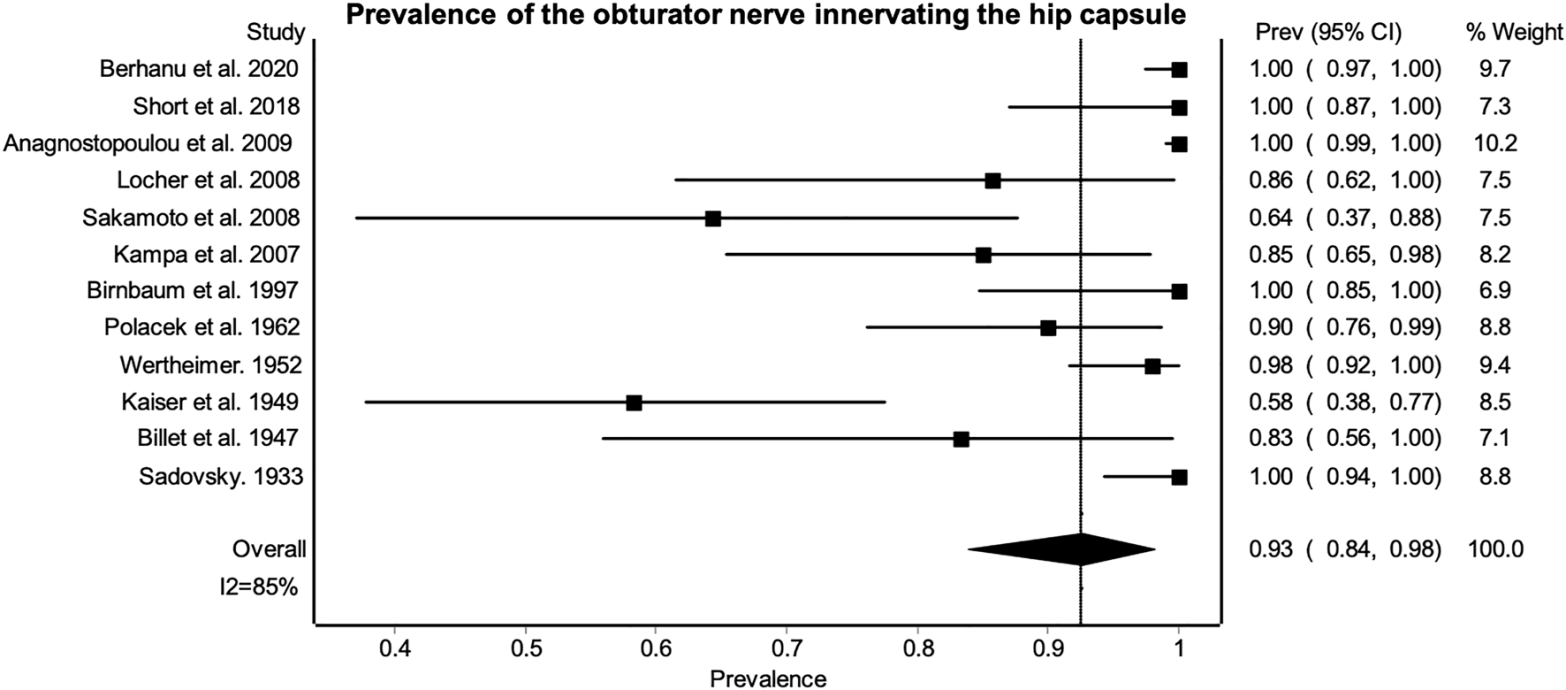
Prevalence of the obturator nerve innervating the hip capsule. Results indicate the variation in prevalence across studies with 95% confidence intervals. There is high heterogeneity between studies as shown by the I^2^ statistic of 85%.

**Figure 3:**
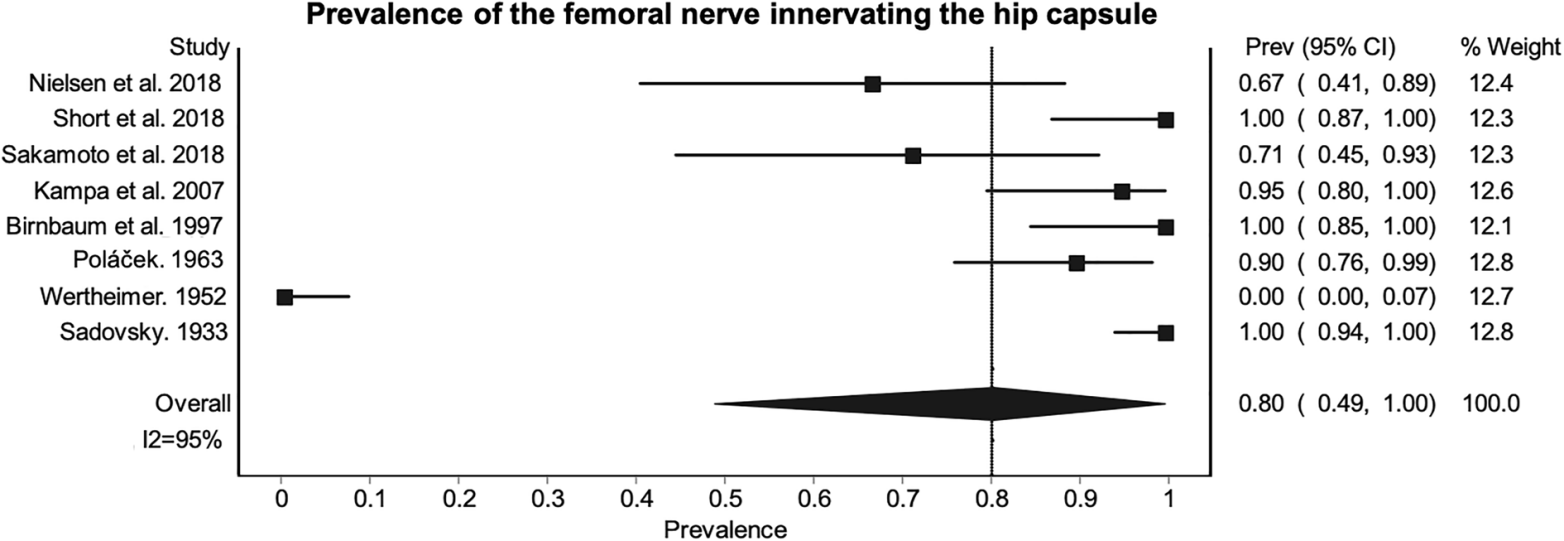
Prevalence of the femoral nerve innervating the hip capsule. Results indicate the variation in prevalence across studies, as well as the high heterogeneity as shown by the I^2^ statistic of 95%.

The NQF appears to be the most frequent nerve to innervate the HC, followed by the obturator then the femoral nerve (Table 4). The AON appears to be present in 15% [95% CI (0.09–0.22), I^2^ = 73%] of the specimens studied in this review^60,67,68,70,77,79,80,83,85^. When the AON is present the PPE that it innervates the HC is 85% [95% CI (0.58–1.00), I^2^ = 86%]^60,67,68,70,77,79,80,83,85^. The prevalence of the AON innervating the HC in the specimens included in the review is presented in Table 4. Innervation of the HC from the AON and obturator nerve appears to be unrelated and highly variable^55,60,67,79,80,83^. The reported PPE of the obturator and femoral nerves are limited, as some studies which reported absence^55^ and presence of articular branches^58,59,61^ did not indicate the number of samples studied, so could not be included in the PPE (Figs. 2 and 3).

**Table 4:** Prevalence of nerves innervating the hip capsule.

Nerve	Number of studies	Total sample size	Prevalence (%)	I^2^	LFK index
Accessory femoral nerve	2	41	27 (95% CI [0.15–0.42])	0	NS
Accessory obturator	9	1225	12 (95% CI [0.07–0.18])	67	-0.03
Femoral	8	156	80 (95% CI [0.49–1.00])	95	-0.67
Inferior gluteal	4	116	2 (95% CI [0.00–0.06])	36	2.07
Nerve to quadratus femoris	2	73	99 (95% CI [0.97–1.00])	0	NS
Obturator	11	441	93 (95% CI [0.84–0.98])	85	-3.85
Sciatic	6	185	50 (95% CI [0.00–1.00]	98	2.07
Superior gluteal	3	61	57 (95% CI [0.25–0.87])	82	0.99

The LFK index of the accessory femoral nerve and nerve to quadratus femoris could not be calculated as only two studies were included in their meta-analysis of prevalence.

*CI* confidence interval, *LFK index* Luis–Furuva–Kanamori index, *NS* not stated.

Considerable heterogeneity was present between studies, except for the AFN and NQF, which had low heterogeneity and the inferior gluteal nerve, which has low-moderate heterogeneity, as indicated by the high I^2^ statistic. The AON also had substantial heterogeneity. High publication bias was present within the meta-analysis for the obturator, sciatic and inferior gluteal nerves as represented by the major asymmetry in the LFK index. As a result of considerable heterogeneity and LFK index, the confidence intervals of the prevalence should be considered. The confidence intervals show that there is minimal variation in the PPE for the AON, NQF and inferior gluteal nerves, but these are based on few studies with small sample sizes. It is unclear if these reported PPE’s are more reliable. Furthermore, PPE specific innervation patterns across the whole HC could not be determined as limited data was available. The NQF and obturator nerves appear to be most prevalent, but the reported figures may not be representative of the wider population.

### Most articular branches to hip capsule are located anteriorly

A variable number of articular branches innervate the HC from each nerve (Table 5). These articular branches were described as thin, small and delicate structures^54,57,58,62,72,77–79,82,87^, which measure up to 17 μm in diameter^78^.

**Table 5:** Articular branches of the hip capsule.

Nerve	Range of branches	Average maximum number of branches	References
AON	0–7	3.2	^63,83,85,86,89^
Femoral	0–14	4.75	^62,67,77,83^
Inferior gluteal	0–4	4	^83^
NQF	0–5	3.6	^76,83,87^
Obturator	0–7	3.33	^54–56,62,67,69,77,82,83^
Sciatic	0–4	3	^62,83^
Superior gluteal	0–4	2.5	^62,83^

This table displays the range of branches and average maximum number of branches noted. Insufficient information prevented the average number of branches or information on the accessory femoral nerve from being displayed.

*AON* accessory obturator nerve, *NQF* nerve to quadratus femoris.

Further detailed information was provided for the obturator nerve but no other nerve, its articular branches span the HC in a 7–38 mm band, which appears to be wider medially^69^. Based on the density of innervation, the HC appears to receive greater innervation from nerves located anteriorly.

### All regions of the hip capsule receive innervation from the lumbosacral plexus

**Figure 4:**
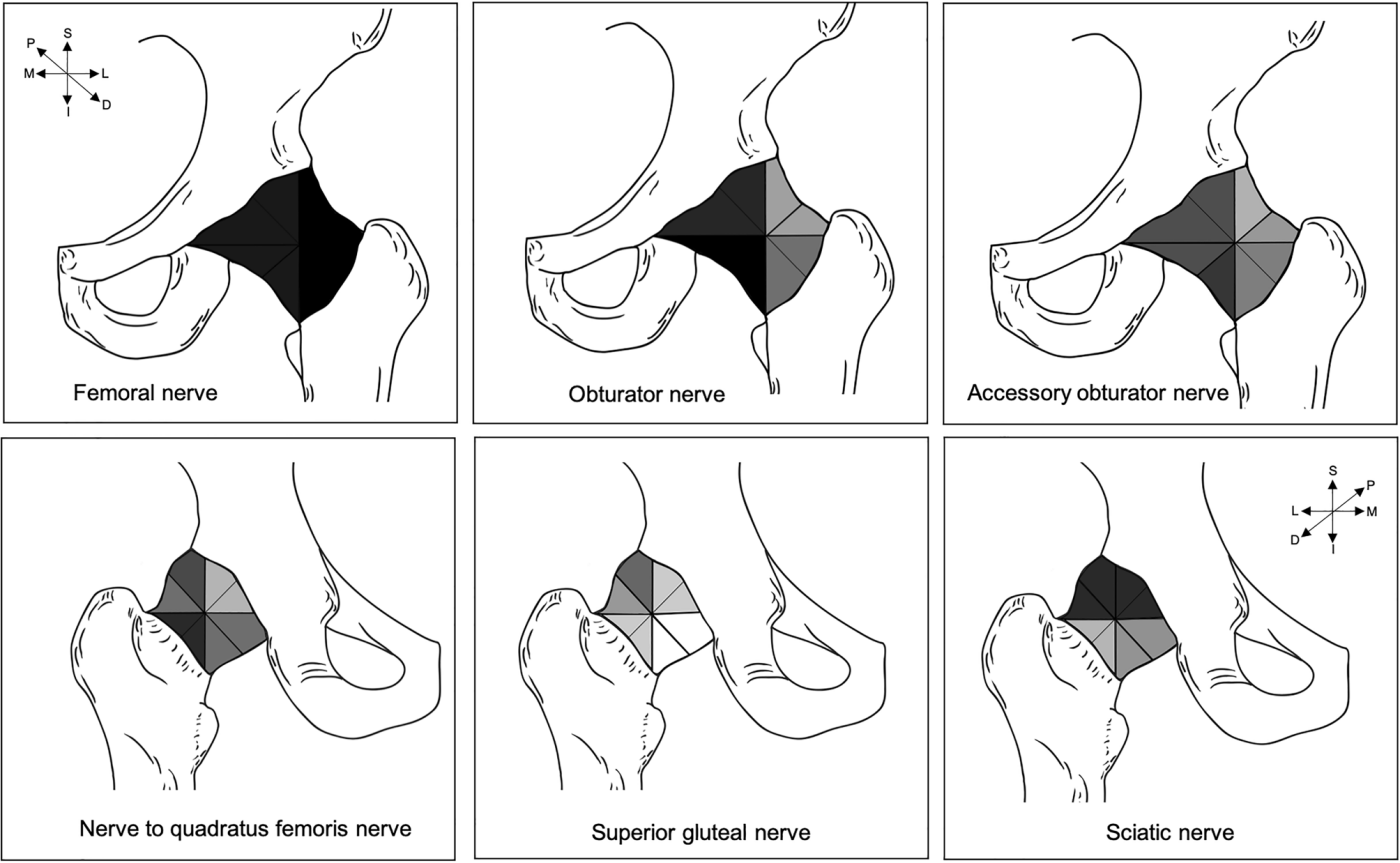
Sketch summarizing consensus findings on the distribution of innervation patterns of the accessory obturator, femoral, obturator, nerve to quadratus femoris, sciatic and superior gluteal nerves. Sketch adapted from Tomlinson et al (2020)^19^. The top row of sketches represents the anterior capsule and the bottom row shows the posterior capsule. Insufficient information was available on the distribution of innervation from other nerves of the lumbosacral plexus; therefore, they were not included in this diagram. The intensity of the shading relates to the percentage of studies that agree that a particular region of the hip capsule receives innervation from a certain nerve. Darker regions depict areas where the literature demonstrates more general consensus that the region is innervated by the nerve. Lighter areas are regions of less general consensus that the regions are supplied by a particular nerve. *D* distal, *I* inferior, *L* lateral, *M* medial, *P* proximal, *S* superior.

More information is available on the distribution of the obturator and femoral nerve than other nerves that innervate the HC. Both of these nerves supply the anterior hip capsule (AHC). Distribution of the branches from the femoral nerve appears to be nearly homogenous across the AHC in comparison to other nerves (Fig. 4). Strong general consensus indicates that the obturator nerve and AON innervate the anterior medial HC, with the articular branches of the obturator nerve commonly present over the radiographic teardrop of the inferomedial acetabulum^67^. General consensus indicates that the AFN supplies the AHC^56,76,77^, but no study to date provides more detailed information on the distribution of the AFN. Furthermore, one study which studied the HC’s innervation in a clockface orientation noted an absence of innervation in the anterior superior aspect of the HC at around the one o’clock to half two position^83^. However, other studies did not study the HC in this detail, nor did they report an absence of innervation in this region.

The posterior capsule appears to be supplied by the superior gluteal, inferior gluteal, sciatic nerves, NQF and also the femoral nerve^58,87^ in a few cases. More specifically, general agreement indicates that the sciatic nerve supplies the posterosuperior capsule^55,61,76,83^ and the NQF innervates the posterior inferolateral portion^76–78,83,87^. The posteroinferior portion of the HC seems to be supplied by the inferior gluteal nerve^83^, however, this is based on a couple of samples in one relatively small-sample anatomical study (n = 20). Current literature remains unclear as to whether there are different innervation patterns and if any variables may predict these patterns.

### Information on the hip capsule’s innervation according to demographic, anthropometric variables and pathology is lacking

To date, no studies have made an attempt to quantify differences in the HC’s innervation according to demographic or anthropometric variables. Nor have these studies stated if individuals with pathology were included in their sample. Furthermore, the details of demographic and anthropometric variables of included cadavers were not routinely collected or at least not reported in detail. Based on the limited information, no differences appear to be present as a result of age^58,60,63^, however, no study has made attempt to compare this directly. A few studies investigated both sides of the HC^68,70,71,73,80,82–85,89^, one of which noted individual differences in the innervation pattern between sides, but omitted to compare this statistically^57^. It remains unclear, if there are differences in prevalence, density, distribution of innervation according to ethnicity, age, sex or other variables.

## Discussion

The primary aim of this systematic review and meta-analysis was to provide an encompassing synthesis on the macroscopic studies investigating the HC’s innervation. Several key findings have been identified from this review, most notably that specific information on the innervation of the HC is lacking. There was high heterogeneity between studies, therefore the presented PPE and distribution patterns may not be applicable to all individuals.

### Articular branches to the hip capsule originate from many nerves of the lumbosacral plexus

Strong evidence indicates that the HC is supplied by a variety of branches of the lumbosacral plexus, primarily by articular branches from the NQF and obturator nerve. There is however considerable heterogeneity between studies. The PPE’s have many limitations as they are formed from studies with different methodologies and some are based on only a few studies with small sample sizes particularly for the AON, NQF and inferior gluteal nerve. Consequently, it seems likely that studies employing larger sample sizes or by different examiners may produce different results in the future. On the other hand, numerous articular branches were noted making it unlikely that dissections have been consistently misinterpreted.

Other nerves including the femoral, superior gluteal and sciatic may contribute to HC’s innervation in some individuals. However, this is uncertain as the root of these articular branches were not traced proximally and histological evaluation has not been performed to date. Rarely, the HC is supplied by other nerves of the lumbosacral plexus. Primarily, this highlights that injuries to the NQF and obturator nerve should be crucially avoided during THA and these nerves should be targeted during RFA in order to reduce pain. Clinicians should consider that articular branches from other nerves provide substantial nerve supply to the HC, therefore patients may continue to have some pain following RFA.

### The inferior and medial regions of the posterior hip capsule seem the most suitable areas for capsular incision during THA, in order to reduce the risk of injuring articular nerves

The anterior inferomedial region appears to have the greatest abundance of nerve supply, but all regions of the HC appear to be innervated. These findings do not completely corroborate with findings from a recent review of microscopic studies^44^, which reported that the superolateral region had the majority of mechanoreceptors. This indicates that the nerve may not terminate as type I–III mechanoreceptors in the same region as where the nerve enters the HC at the gross level, i.e., macroscopically visible nerves may have an intra-ligamentous course within the HC. When considering the macroscopic and microscopic innervation of the HC, the inferior and medial regions of the posterior HC appear to receive the least innervation. Risk of damaging articular nerves may therefore be lowest when incising these regions during THA. THA via the posterior approach with capsular repair may be most appropriate for the non-risk patient in order to reduce the risk of disrupting neural feedback loops, which could contribute to lower post-operative hip joint dislocation rates. Alternative approaches should continue to be considered for at-risk patients, i.e. those of older age, with a BMI of > 30 and with mobility or neuromuscular pathology^38^. The recent literature is contradictory with regards to which surgical approach produces the highest rate of instability post-operatively^36–41^. Therefore, implementing a combination of best practices which reduce the risk of dislocation may be more important when planning surgery than the surgical approach alone, including repairing the HC^8–10^ to restore biomechanical stability, preserving musculature and other surrounding soft tissue^8^, as well as correct joint alignment^9,35^.

The anterior approach during THA appears most appropriate to reduce pain post-operatively, as the current literature reports lower patient pain scores following this approach^90^. The literature on microscopic innervation of the HC provides limited evidence with regards to the distribution of FNEs^44^. However, the capsule clearly appears to have a role in nociception and more robust immunohistochemical experiments mapping the capsule will allow more solid conclusions and recommendations to be formed.

Furthermore, further research is required to determine the course of nerves towards and across the surface of the HC, as well as the differences in the number of nerves and terminal endings present in the surrounding tissues. Recent research highlights that the hip capsule may have the least innervation in comparison to the different tissue layers of the hip joint, including the skin, subcutaneous fat, muscle and fascia^91^. It may therefore be more important to consider the innervation of all of the soft tissue layers, variation between individuals and feasibility to avoid large nerve bundles when planning surgery. A comprehensive map of the nerves and end-terminals within all tissue layers surrounding the hip joint is lacking to date. Additionally, it is important to avoid vessel damage during THA as in doing so it is likely clinicians will also avoid damaging nerves, this supports previous microscopic findings^44^. The current literature suggests the HC should be repaired following THA, in order to reduce dislocation risk and allow for neural repair. When considering regions to target during RFA, the anterior inferomedial region appears to be a suitable location. However, further study is required that considers the cumulative density and distribution of articular branches in each region, as well as the modality of these nerves.

### The origin of the capsular nerve branches resembles the innervation pattern of the hip muscles

Many of the articular branches are derived from branches to musculature, rather than the common trunk of the nerve. These findings align with Hilton’s law^45^ and suggest that these muscles may contribute to proprioceptive control of the joint in response to changes in tension of the HC. To date, no study has recorded the origin of articular nerves and their relationship to musculature that acts on the joint, as a result the risk of nerve damage during THA and RFA is unknown. The literature also highlights that during pelvic surgery near the obturator canal there is considerable risk that both the sensory supply to the HC and the motor innervation to the muscles surrounding the joint could be damaged. Nerve damage is unlikely during RFA, as sensory testing is commonly performed prior to lesioning and some practitioners check for absence of motor stimulation^4^. Muscular weakness following RFA has not been commonly reported, however, this may be as muscular strength before and after RFA are not standardly recorded^4^. Albeit rare, these complications have been noted following THA due to insufficient muscular fixation^34^, muscular or nerve damage^92,93^. This can result in dislocation^34^, falls, numbness, muscle palsy or pain^92^. The rates of muscular weakness appear to differ according to the approach used^30,32^ or the experience of the surgeon^94,95^. It remains unclear which approach has the lowest risk of functional impairment^31,33^. Nonetheless, most of these nerve palsies appear to recover within 1–3 years^92,93^ and muscular weakness seems to improve with physiotherapy^30^ or further surgery^96^. Considering this, clinicians should take care when performing RFA and surgical procedures to avoid damaging nerves, periarticular soft tissue and muscles in order to preserve potential proprioceptive and nociceptive functioning. Additionally, where possible, surgeons should repair the HC and overlying muscles. Furthermore, increased accuracy during these procedures may be possible with intra-procedural imaging, such as polymetric imaging during THA^97^ or visualisation of RFA lesions using near field ultrasound^98^. Additionally, it may be beneficial to measure motor strength of the muscles related to the targeted nerves both pre and post intervention in order to identify individuals at increased risk of dislocation as a result of potential damage to neural feedback loops. It is important to gain greater understanding of the innervation of the HC and associated muscles to aid clinicians in preserving function of the hip joint. Further research should employ techniques that minimise the risk of spatial distortion or damage to the articular branches to increase the accuracy of the description of these branches.

### Differences in innervation of the hip capsule appear to be present between individuals

No information is available with regard to the relationship between demographic and anthropometric variables and their effect on HC innervation, but differences have been observed between individuals. This is partially due to incomplete descriptions of the sample characteristics limiting possible comparison between studies. The high heterogeneity reported between studies highlights that subgroup analysis should be performed in future research. It is important to understand the differences in innervation according to demographic variables as this may highlight that an individualized approach to surgery may be required. Male patients are at greater risk of revision following THA due to dislocation, particularly when the posterolateral approach is employed^42^. Whereas, age of the patient did not appear to alter the risk of revision due to dislocation^42^. Current systems available to adapt THA to address the sex-specific differences in boney morphology, such as the femoral head size and neck offset are thought to be sufficient to produce satisfactory outcomes and reduce this risk^99^. However, procedural changes according other demographic variables, such as ethnicity may be required. There are differences in the arrangement of the lumbar plexus according to ethnicity^100^. These alterations to innervation patterns may also relate to HC innervation, but this has not been studied to date.

Differences have also been noted in microscopic innervation between individuals according to age^101^ and pathology^1^. Specifically, previous microscopic investigation showed a decreased density of mechanoreceptors in the HC of individuals with osteoarthritis compared to those with no known pathology^1^. However, it is remains unknown if these differences may be observed macroscopically, as it has not been studied to date. Further studies should include larger sample sizes in order to substantiate the differences in HC innervation according to demographic, anthropometric variables or pathology (e.g. sex, side, age, pelvic dimensions). This information may increase understanding why there is conflicting evidence on which approach produces lower dislocation rates as different approaches may be required during THA or RFA in specific patient populations.

### Articular nerve density, distribution and prevalence of capsular branches seem highly dependent on the quality of the studies in current literature

The reported data on HC innervation are at risk of invalid measurement, as these are highly dependent on method employed to visualise these nerves. Numerous biases are present within the available literature that will affect the given summary of results in this review and meta-analysis. Firstly, a substantial number of studies were retrospective studies and case reports, which therefore introduces the risk of selection bias, detection and publication bias. In order to reduce the effect of this bias, these studies were excluded from the meta-analysis. Secondly, no study stated that more than one author reviewed the structures identified. Thirdly, it is possible that the articular branches may have been misidentified, as limited information is available on the methods employed, which prevents the quality of the work from being assessed. Fourthly, the PPE reported is based on a small number of studies. It is possible that the reported prevalence may differ in the wider population.

Under-reporting of HC innervation may have occurred as the small articular branches may be damaged easily during dissection^69^. To decrease the risk of under-reporting, several authors used magnification to aid in identifying these structures, one of which deemed this necessary^77^. Certain embalming methods reduce the risk of articular branches being damaged as they make structures more easily identifiable and forms the surrounding tissue in a suitable consistency for blunt dissection. Ethanol-based^102^, phenoxyethanol or Thiel^103^ embalmed cadavers may be more suitable for performing future macroscopic studies of nerves, ligaments and muscles in terms of haptics, compared to the stiffer tissues resulting from formaldehyde and ethanol embalming^104^. These specific embalming methods have not been employed to date when assessing HC innervation, this is likely because of the known regional differences in anatomical departments which favor one special embalming technique. Authors should consider the fixation of cadavers used in future work thoroughly as Thiel embalming does not permit for histologic assessment of small nerves^104^.

Inaccurate reporting of HC innervation may be present in the current literature, as to date no study has used dyes, nor confirmed their findings with histological analysis. It may be possible that structures were misidentified. There are inconsistencies in the current literature with regards to the methodologies employed, for example in the use of magnification and embalming technique used. This highlights many areas for potential errors in the reporting of results. Additionally, identifying these inconsistencies allows future work to be more robust. However, the quality of anatomical dissection studies is often criticised and many of the limitations are unavoidable and a product of dissection studies. Supplementary investigations, such as histology, immunohistochemistry, or Sihler’s staining which aid in the identification of nerves would be extremely beneficial and would add credibility to the findings on HC innervation.

### Limitations

Difficulties were presented by performing meta-analysis of gross anatomical studies, as methodologies and sample sizes were variable, few studies could be compared, and several factors increased the risk of invalid reporting. This is clearly represented by the high heterogeneity reported between the studies included.

It is possible that suitable articles may have been missed through the application of exclusion criteria, search filters, limited keywords searches and articles whose full text could not be located. However, to minimise the risk of bias and underreporting of the literature, articles in all languages were included if accessible to the authors. Some bias may persist as unpublished work was excluded, however, this was recommended previously^50^, as these may have not undergone a rigorous peer review. Finally, location bias may affect the results as articles in less accessible journals may have been missed by our search criteria. To address this issue, a variety of databases was searched.

## Conclusion

The gross anatomical innervation of the HC seems highly variable between individuals. Based on the current literature, the authors recommend that RFA procedures should continue to target the anterior inferomedial aspect of the HC but remain aware that other nerves are spared during this procedure and this may not completely resolve pain. In order to spare articular nerves and potential proprioceptive functioning during THA, the posterior approach appears to be most appropriate for non-risk patients. The HC should be repaired following THA as this is proven to lower dislocation risk and may aid in restoring HC innervation. Surgeons should continue to consider other factors which reduce dislocation risk when planning surgery. A coherent map of HC innervation is required to be able to improve the patient’s prognosis following RFA and THA. This research should use additional investigations to support dissection findings, such as microscopy.

## Data Availability

The articles included in this systematic review and meta-analysis may be found using the search strategy outlined in the methods section.
